# Endomembrane H-Ras Controls Vascular Endothelial Growth Factor-induced Nitric-oxide Synthase-mediated Endothelial Cell Migration*

**DOI:** 10.1074/jbc.M112.427765

**Published:** 2013-04-02

**Authors:** Dagmar J. Haeussler, David R. Pimentel, Xiuyun Hou, Joseph R. Burgoyne, Richard A. Cohen, Markus M. Bachschmid

**Affiliations:** From the ‡ Vascular Biology Section and; § Myocardial Biology Unit, Whitaker Cardiovascular Institute, Boston University School of Medicine, Boston, Massachusetts 02118 and; ¶ Cardiology, King's College London, London SE1 7EH, United Kingdom

**Keywords:** Angiogenesis, Cell Migration, Nitric Oxide, Nitric-oxide Synthase, Protein Palmitoylation, Vascular Endothelial Growth Factor (VEGF), H-Ras, Endomembrane

## Abstract

We demonstrate for the first time that endomembrane-delimited H-Ras mediates VEGF-induced activation of endothelial nitric-oxide synthase (eNOS) and migratory response of human endothelial cells. Using thiol labeling strategies and immunofluorescent cell staining, we found that only 31% of total H-Ras is *S*-palmitoylated, tethering the small GTPase to the plasma membrane but leaving the function of the large majority of endomembrane-localized H-Ras unexplained. Knockdown of H-Ras blocked VEGF-induced PI3K-dependent Akt (Ser-473) and eNOS (Ser-1177) phosphorylation and nitric oxide-dependent cell migration, demonstrating the essential role of H-Ras. Activation of endogenous H-Ras led to recruitment and phosphorylation of eNOS at endomembranes. The loss of migratory response in cells lacking endogenous H-Ras was fully restored by modest overexpression of an endomembrane-delimited H-Ras palmitoylation mutant. These studies define a newly recognized role for endomembrane-localized H-Ras in mediating nitric oxide-dependent proangiogenic signaling.

## Teaser

**Background:** Subcellular localization modifies H-Ras signaling.

**Results:** In H-Ras-ablated cells, endomembrane-delimited H-Ras fully reconstitutes vascular endothelial cell growth factor (VEGF)-induced phosphatidylinositol 3-kinase (PI3K)/Akt/endothelial nitric-oxide synthase (eNOS) signaling, eNOS activity, and endothelial cell migration.

**Conclusion:** Endomembrane H-Ras is sufficient for proangiogenic VEGF signaling.

**Significance:** Novel endomembrane H-Ras signaling via PI3K/Akt/eNOS constitutes endothelial angiogenic signaling.

## Introduction

The small GTPase p21Ras is a major molecular switch, regulating various cellular responses, including proliferation, survival, migration, and apoptosis. After binding GTP, Ras undergoes a conformational change and activates downstream signaling partners by recruiting them to distinct cellular membrane locations. Thus, activation through Ras occurs by interaction with effector Ras-binding domains (RBDs),^2^ such as that found in Raf and the catalytic subunit p110 of phosphatidylinositol 3-kinase (PI3K) (1). Ras activity is regulated by GTPase-activating proteins that render the protein GDP-bound and thus inactive and guanosine exchange factors that facilitate the GDP/GTP exchange, which activates Ras.

Despite a substantially differing hypervariable region at the C terminus, all three isoforms (H-Ras, K-Ras, and N-Ras) share high sequence homology. Principally, Ras isoforms can interact with the same down-stream effectors, but isoform-specific signaling events are regulated by the expression profile and localization to discrete cellular membranes and microdomains (2, 3). Successful plasma membrane (PM) localization of H-Ras requires at least two different types of post-translational modifications at the C terminus. Farnesylation of cysteine 186, a modification of the CAA*X* motif and common to all isoforms, weakly tethers Ras to the endoplasmic reticulum. There, the AAX is proteolytically cleaved, and the terminal farnesylated Cys-186 is methylated. In H-Ras, this weak membrane interaction is strengthened by palmitoylation of Cys-181 and Cys-184 in the hypervariable region, resulting in a stable PM localization (4, 5).

At the PM, the thioester bond is hydrolyzed, relocalizing H-Ras to endomembranes, including the Golgi and endoplasmic reticulum (5, 6). The molecular mechanism of H-Ras depalmitoylation, the key enzymes involved, and their regulation are still under investigation (7–9). Despite the long held belief that H-Ras signals exclusively from the PM and PM-derived endosomes (10, 11), constitutively active H-Ras may mediate GTPase signaling at endomembranes, especially the endoplasmic reticulum and Golgi, and two recent publications have demonstrated that this phenomenon occurs with endogenous H-Ras in cell lines (12, 13). To date, functional studies in primary cells have not been performed, and it is unknown whether or not endomembrane H-Ras is involved in physiological endothelial cell (EC) signaling.

For angiogenesis in the adult, the importance of H-Ras signaling is well recognized (14–17). VEGF is a central modulator of angiogenesis in ECs, and its signaling via H-Ras modulates EC function. However, the molecular mechanism through which the VEGF receptor transmits the signal to this isoform remains elusive. VEGF stimulation increases Grb2 association with the receptor (18) and increases Ras GTP loading. However, in ECs, VEGF-induced Raf/MEK/ERK activation is mainly mediated by PKC (19, 20). Nevertheless, GTP-bound H-Ras associates with various downstream effectors important for angiogenesis, including Raf1 and PI3K, which in turn mediate proliferative, migratory, and survival signaling.

Using thiol labeling strategies, we found that 31% of H-Ras was palmitoylated in human aortic endothelial cells (HAECs), leaving the function of the large majority of H-Ras in the cell unexplained. In this study, we demonstrate an essential role of endomembrane H-Ras in EC signaling. By using a non-constitutively active palmitoylation-deficient H-Ras mutant that solely localizes to endomembranes, we found that activation of H-Ras at endomembranes is sufficient for VEGF-mediated signaling that controls the angiogenic response via PI3K, Akt, and eNOS phosphorylation, leading to EC migration.

## MATERIALS AND METHODS

### 

#### 

##### Cell Treatments

EC studies were performed with HAECs (Lonza), cultured in complete endothelial growth medium (EGM-2, Lonza) supplemented with 1% penicillin, streptomycin (Invitrogen) and serum-starved overnight in basal endothelial medium (EBM-2 containing 0.1% FBS, Lonza) supplemented with 1% penicillin, streptomycin before experiments. HAECs between passages 8 and 11 were used for all experiments.

Knockdown of endogenous H-Ras was performed with siRNA (Dharmacon) designed against the 3′-untranslated region (ACAGAUGGGAUCACAGUAAUU) and Lipofectamine 2000 (Invitrogen). For ablation of endogenous N-Ras, we used pooled siRNA (Dharmacon, siGENOME, M-003919-00-0005). The validity of the knockdown was assessed after 48–62 h. For efficient knockdown, 90 pmol/ml of siH-Ras and 45 pmol/ml siN-Ras were used. Targeting the 3′-untranslated region of H-Ras allowed for concomitant adenoviral overexpression of H-Ras mutants. Knockdown did not affect cell viability, which was at 97 ± 1.3% for siControl and 98 ± 0.5% for siH-Ras.

H-Ras vectors were generated by subcloning pCMV H-Ras (Clontech) into pcDNA3.1/His-XPRESS (Invitrogen). The palmitoylation-deficient mutant H-Ras PalM was generated by mutating cysteines 181 and 184 to serines using site-directed mutagenesis (Stratagene), and adenoviral vectors were made utilizing the AdEasy system (QBiogene). Adenoviruses were then amplified from single clones, purified according to the manufacturer's protocol, and screened for sequence integrity and the absence of contaminating viral E1A recombination as described previously (21). HAECs were infected with adenoviruses for 48 h before performing assays. After treatment, cells were rinsed with PBS before being lysed into either Laemmli buffer, the respective buffer for measuring eNOS enzyme activity, buffer for enrichment of palmitoylated protein, or fixed in 4% paraformaldehyde for imaging.

##### RNA Isolation and Quantitative PCR

RNA was isolated using TRIzol (Invitrogen) according to the manufacturer's protocol. RNA was resuspended in RNase/DNase-free water (Fisher), and concentration was measured at 260 nm using a NanoDrop spectrophotometer (Fisher). cDNA was generated by reverse transcription PCR from 1 μg of RNA using the high capacity RNA to cDNA kit (Applied Biosystems).

Quantitative PCR was performed using FAM/NFQ-conjugated TaqMan inventoried assays from Applied Biosystems for human H-Ras (Hs00610483_m1), K-Ras (Hs00270666_m1), and N-Ras (Hs00180035_m1) and murine H-Ras (Mm00476174_m1), K-Ras (Mm00517491_m1), and N-Ras (Mm03053787_m1). The VIC/MGB-labeled β-actin primer/probe set (human, 4326315E; mouse, 4352341E) was used as an internal control, and results were normalized to this *Ct* value. Quantitative PCR was performed using a StepOnePlus real-time PCR instrument (Applied Biosystems) with the following program: 10-min initial denaturation at 95 °C, followed by 40 cycles of denaturation at 95 °C/15 s and annealing/extension at 60 °C/1 min. ΔΔ*Ct* analysis was performed.

##### Wound Healing/Scratch Assay

Wound healing assays were performed in confluent and quiescent HAECs. All treatments were performed in basal medium containing 0.1% FBS at the time of the scratch, unless otherwise stated. In order to injure the intact monolayer of ECs, a scratch was made with a sterile pipette tip, and pictures were taken at 0 and 16 h at three defined locations of about 15–25 cells each (*n* = 1). The migration distance was assessed using ImageJ software (National Institutes of Health) as described previously (22). After migration, cells were collected in Laemmli buffer, and knockdown and overexpression were confirmed for each experiment.

##### Affinity Capture of Palmitoylated Proteins

Palmitoylated proteins were affinity-captured as described previously with some modifications. A confluent 100-cm dish with HAECs was either lysed in reducing, denaturing buffer (20 mm DTT, 1% SDS, 100 mm Tris, pH 7.4) and incubated for 20 min at room temperature to reduce all cysteines or lysed in blocking buffer (100 mm maleimide, 1% SDS, 100 mm Tris pH 7.4) and incubated for 20 min at 50 °C to alkylate all free cysteines. Excess DTT or maleimide was removed using Zeba desalting spin columns (Pierce). For specific thioester hydrolysis and cysteine labeling, the samples were supplemented with 400 mm hydroxylamine, 167 μm HPDP-Biotin (Pierce), 0.5% SDS and incubated for 1 h with agitation at room temperature. Hydrolysis and labeling reactions were terminated by a second desalting step. Biotinylated proteins were purified with magnetic streptavidin-agarose beads (Pierce). The agarose beads were washed four times with PBS containing 0.5% Tween 20, and HPDP-Biotin-modified protein was eluted by boiling with Laemmli buffer (5% 2-mercaptoethanol). The input before and after streptavidin agarose pull-down was resolved on 15% SDS-polyacrylamide gels. Band intensities were measured using ImageJ. A schematic depiction of the method is included in Fig. 3*A*.

##### Immunoblotting

Purified proteins following the hydroxylamine-dependent biotin switch assay, eNOS activity assay or unprocessed cell lysates were separated on SDS-polyacrylamide gels and transferred to polyvinylidene fluoride membranes (Immobilon, Millipore), blocked in 5% nonfat milk, and incubated overnight with the respective antibodies. Immunostained membranes for H-Ras (catalog no. ab32417, Abcam), Ras10 (catalog no. 05-516, Millipore), LacZ (β-galactosidase, catalog no. ab616, Abcam) and GAPDH (catalog no. sc25778, Santa Cruz Biotechnology, Inc., Santa Cruz, CA) were incubated with IR dye-conjugated secondary antibodies and visualized using the LI-COR Odyssey system. Membranes probed for phosphorylated (Ser-473, catalog no. 9271, Cell Signaling) and total Akt (catalog no. 2920, Cell Signaling), phosphorylated (Thr-202, Tyr-204, catalog no. 9101, Cell Signaling) and total ERK (catalog no. 9102, Cell Signaling), phosphorylated (Ser-1177, catalog no. 612392, BD Biosciences) and total eNOS (catalog no. 610296, BD Biosciences), and phosphorylated (Ser-239, catalog no. 3114, Cell Signaling) and total VASP (catalog no. 07-1288, Millipore) were developed using HRP-conjugated secondary antibodies and visualized using ECL (GE Healthcare). Band intensities were measured using ImageJ, and values were normalized to GAPDH as a loading control.

##### Immunohistochemistry

HAECs for immunohistochemistry were grown to 60–80% confluence on polylysine (Sigma)-coated 4-well chamber slides (Nunc). After treatment, cells were rinsed with PBS and fixed in 4% fresh paraformaldehyde for 10 min. Subsequently, cells were washed three times with PBS and permeabilized in 1 mg/ml saponin for 10 min. Cells were blocked for 1 h in 5% IgG and protease-free BSA (Jackson ImmunoResearch) at room temperature and after another three washes incubated overnight at 4 °C in primary antibody supplemented with 0.5% BSA. Unless otherwise stated, overexpressed XPRESS-tagged H-Ras was stained with XPRESS antibody (1:400, Invitrogen), and phospho-eNOS was stained with an antibody against Ser-1177 (1:50, catalog no. 612392, BD Biosciences). The next day, cells were rinsed three times in PBS and then incubated with the secondary antibody anti-mouse Alexa Flour 594 (1:500 in 0.5% BSA) for 1 h at room temperature before being washed another three times. Mounting medium containing DAPI stain (ProLong Gold, Invitrogen) was placed onto the surface of each slide, followed by a coverslip that was then sealed using nail polish. Slides were viewed using an Olympus DSU confocal microscope to determine subcellular distribution of overexpressed H-Ras. A fluorescence (Nikon Eclipse 80i) microscope was also used to image subcellular distribution of eNOS phosphorylation upon stimulation.

##### Nitric-oxide Synthase Activity Assay

eNOS activity was measured by separating ^14^C-labeled l-arginine and l-citrulline on thin layer chromatography (TLC) plates as described by Kumar *et al.* (23). In brief, HAECs treated as indicated were lysed in PBS containing 1% Nonidet P-40 alternative (Millipore), 25 mm NaF, and complete mini protease inhibitor mix (Roche Applied Science). After measuring protein concentration (protein assay, Bio-Rad), 20 μg of protein was added to the ^14^C-labeled l-arginine-containing reaction mix (23) and incubated at 37 °C for 30 min. The reaction mix contained the following cofactors: β-nicotinamide adenine dinucleotide 2′-phosphate reduced tetrasodium salt (final 2 mm, Sigma), (6*R*)-5,6,7,8-tetrahydrobiopterin dihydrochloride (final 100 μm, Sigma), CaCl_2_ (final 1 mm, Sigma), flavin adenine dinucleotide disodium salt hydrate (final 100 μm, Sigma), calmodulin (final 0.05 μg/μl, EMD Millipore). All reagents were dissolved in 30 mm Hepes (pH 7). The reaction was terminated by adding 2 volumes of ice-cold methanol, and precipitated protein was removed by centrifugation. The supernatant was spotted onto a silica gel TLC plate (Sigma), and the product was separated from the substrate using ammonium hydroxide/chloroform/methanol/water (ratio 2:0.5:4.5:1). After chromatography, the separation was visualized, and signals were integrated using the Molecular Imager (Bio-Rad) phosphor imager system. To report VEGF-stimulated eNOS activity, l-citrulline production by non-stimulated cells was subtracted from that of VEGF-stimulated cells, and to account for experimental variations, the results are reported as the percentage of l-citrulline from total signal (l-arginine + l-citrulline).

##### Ras Activity Assay

Incubation of lysates with an RBD-GST fusion protein allows for a pull-down of active Ras with glutathione-Sepharose beads. Ras activity assays were performed using a Ras activation kit (Jena Bioscience), according to the manufacturer's protocol. In brief, HAECs treated as indicated were lysed into 1 ml of lysis buffer (per 35-mm dish) containing GDP (100 μm) to quench postlytic GTP loading of Ras. Cleared lysates were incubated for 30 min at 4 °C on a rotary shaker with 40 μl of glutathione-Sepharose beads. The beads were spun down and washed one time with 1 ml of lysis buffer. Bound protein was released by boiling in 30 μl of Laemmli buffer (2× concentrated, 5% 2-mercaptoethanol) for 10 min at 95 °C while shaking. Eluted protein was separated on 15% SDS-polyacrylamide gels. Before transferring the separated proteins, the gel containing the pulled-down protein was cut below the 37-kDa protein molecular mass marker, and only the lower half containing H-Ras was transferred to a PVDF membrane and probed for H-Ras. The upper half of the gel was stained with GelCodeBlue (Thermo Scientific) to visualize RBD-GST above the 37-kDa protein marker to control for even distribution of RBD-GST. The ratio of active H-Ras (pull-down) and H-Ras in the lysate (input) was determined by quantifying the bands using ImageJ.

## RESULTS

### 

#### 

##### VEGF-induced EC Migration Is H-Ras-dependent

**FIGURE 1.**
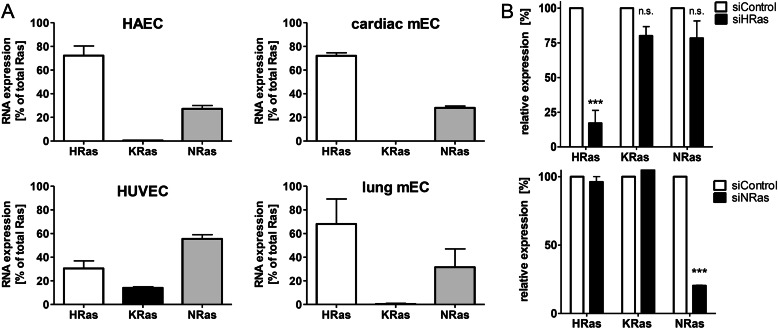
**Relative expression level of the three major Ras isoforms in HAECs and other types of ECs.** *A*, relative expression levels of Ras isoforms in untreated HAECs, HUVECs, cardiac mouse ECs, and lung mouse ECs normalized to β-actin were measured with TaqMan quantitative PCR of mRNA expression levels. *n* ≥ 3. *Error bars*, S.E. *B*, efficacy of H-Ras and N-Ras knockdown. Endogenous H-Ras mRNA was knocked down by targeting the 3′-untranslated region. Relative expression levels of HAECs treated with siControl or siH-Ras are shown. Two-way ANOVA and Bonferroni's post-test were used (*n* ≥ 4). *Error bars*, S.E. ***, *p* < 0.001, siControl *versus* siH-Ras; *n.s.*, not significant.

**FIGURE 2.**
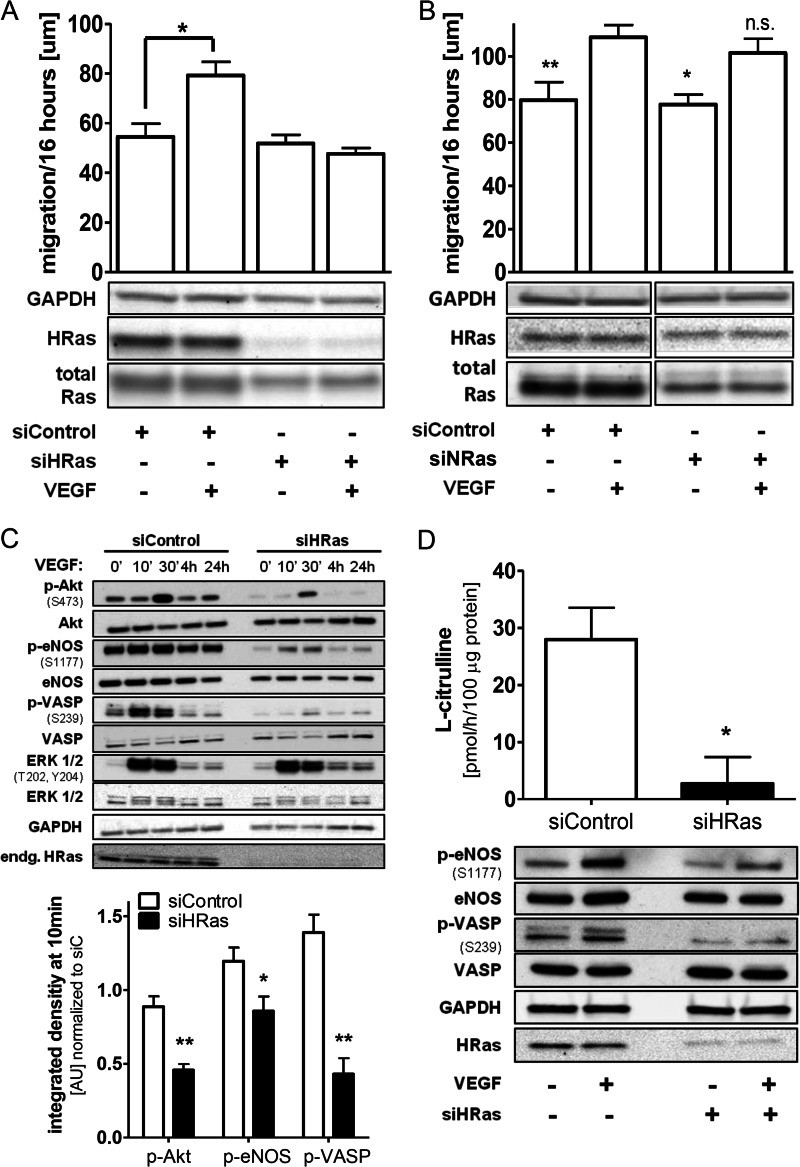
**VEGF-induced EC migration is H-Ras-dependent.** *A*, knockdown of endogenous H-Ras blocks EC migratory response to VEGF. After overnight starvation, a wound healing assay was performed with HAECs using VEGF (50 ng/ml) as a stimulus. One-way ANOVA and Dunnett's post-test were used (*n* ≥ 4). *Error bars*, S.E. *, *p* < 0.05. *B*, knockdown of endogenous N-Ras has no significant effect on EC migration in response to VEGF. A wound healing assay was performed as described in *A*. All depicted bands were on the same membrane and only cut for visual clarity. One-way ANOVA and Dunnett's post-test were used (*n* ≥ 3). *Error bars*, S.E. *, *p* < 0.05; **, *p* < 0.01. VEGF stimulation *versus* no stimulation; n.s., not significant, VEGF-stimulated siNRas *versus* VEGF-stimulated siControl. *C*, knockdown of endogenous H-Ras in HAECs resulted in loss of the key proangiogenic signaling cascade Akt/eNOS, including downstream ^•^NO/PKG effector VASP in response to VEGF. HAECs treated with either siControl or siH-Ras were stimulated after overnight starvation for 0 min, 10 min, 30 min, 4 h, and 24 h with VEGF (50 ng/ml). Densitometric quantification of HAECs treated with either siControl or siH-Ras at 10 min of VEGF stimulation. All values were standardized to GAPDH as a loading control and normalized to siControl at 0 min. Unpaired Student's *t* test was used (*n* ≥ 3). *Error bars*, S.E. **, *p* < 0.01; *, *p* < 0.05. *D*, VEGF-stimulated eNOS activity (Ser-1177 phosphorylation) was reduced by knockdown of endogenous H-Ras. H-Ras-depleted HAECs were stimulated for 30 min with VEGF (50 ng/ml) and lysed. Part of the lysate was processed for Western blotting (*bottom*), and eNOS activity (*top*) was measured *in vitro* by quantifying ^14^C-labeled l-arginine to ^14^C-labeled l-citrulline conversion using a phosphor imager system. Band intensity for basal level was subtracted from VEGF-stimulated eNOS activity. Unpaired Student's *t* test was used (*n* = 4). *Error bars*, S.E. *, *p* < 0.05.

H-Ras is the major isoform in HAECs and in microvascular ECs (murine lung and cardiac ECs), as demonstrated by quantitative RT-PCR of mRNA expression levels (Fig. 1*A*). The role of H-Ras in EC migration was assessed by siRNA targeting of endogenous H-Ras in HAECs. The siRNA specificity toward H-Ras is shown in Fig. 1*B*. Although vascular endothelial cell growth factor (VEGF) (50 ng/ml) led to EC cell migration, H-Ras knockdown abolished the promigratory response to VEGF (Fig. 2*A*).

To ensure that the proangiogenic phenotype is specifically mediated by H-Ras, we depleted HAECs of N-Ras (Figs. 1*B* and 2*B*) and measured EC migration in response to VEGF (Fig. 2*B*). Although N-Ras comprises about 25% of total Ras mRNA in HAECs (Fig. 1*A*), knockdown of this isoform did not alter the migratory response to VEGF, as demonstrated in Fig. 2*B*.

Activated H-Ras binds directly to the RBD of PI3K and triggers the formation of phosphatidylinositol 3,4,5-trisphosphate, which controls Akt activity. Akt via specific phosphorylation of eNOS at position Ser-1177 activates the enzyme and enhances ^•^NO production (24–26). Released ^•^NO increases cyclic GMP and activates cGMP-dependent protein kinase activity (PKG), which increases phosphorylation of VASP at Ser-239 (27, 28). In accordance with the migration data, less Akt (Ser-473), eNOS (Ser-1177), and VASP (Ser-239) phosphorylation (Fig. 2, *C* and *D*) was observed after knockdown of endogenous H-Ras than in control cells after VEGF stimulation, explaining the molecular mechanism for the loss of migratory response. However, VEGF-mediated activation of ERK 1/2 was unaltered after knockdown of the small GTPase, indicating that utilization of siRNA against H-Ras does not influence VEGF receptor behavior *per se*. The levels of total protein of Akt, eNOS, VASP, and ERK 1/2 were not affected by siRNA against H-Ras (Fig. 2*C*).

Consistent with the lack of eNOS phosphorylation at Ser-1177, eNOS activity in H-Ras-depleted cells, measured by conversion of l-arginine to l-citrulline, was attenuated in response to VEGF compared with cells treated with control siRNA (Fig. 2*D*). These data are consistent with a central role of H-Ras in proangiogenic VEGF signaling of HAECs.

##### The Majority of H-Ras Is Not Palmitoylated and Resides at Endomembranes

**FIGURE 3.**
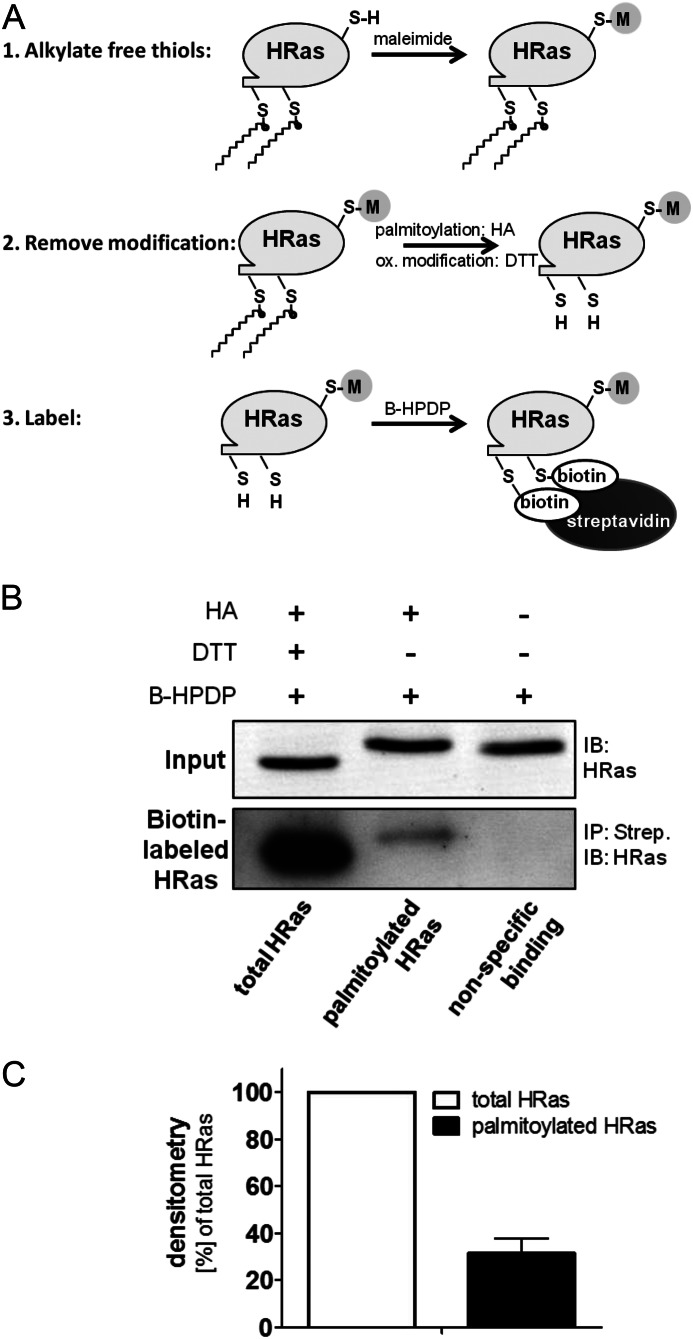
**The majority of H-Ras is not palmitoylated.** *A*, hydroxylamine (HA)-dependent biotin switch is a reversed labeling technique for palmitoylated cysteines. First, free thiols (RSH) are alkylated by maleimide. Then thioester bonds, including palmitoylation, are hydrolyzed by HA, whereas reversible oxidative (*ox*) modifications are removed by DTT. The freed cysteines are subsequently labeled by thiol-reactive HPDP-Biotin (*B-HPDP*), and biotinylated protein is enriched using streptavidin pull-down. *B*, palmitoylation of endogenous H-Ras was measured using the HA biotin switch assay. Lysates were treated either with DTT and HA to label total H-Ras or with HA only to specifically remove *S*-palmitoylation and label freed cysteines with thiol-reactive HPDP-Biotin. The pull-down on streptavidin-agarose (*Strep*) was separated by SDS-PAGE and immunoblotted for H-Ras with an isoform-specific antibody. *IB*, immunoblot; *IP*, immunoprecipitation. *C*, densitometry was performed for streptavidin pull-down (*n* = 3). *Error bars*, S.E.

Thioester-linked palmitoylation of cysteines 181 and 184 tethers H-Ras to the PM, whereas unmodified H-Ras accumulates at endomembranes, preferentially at the Golgi, where palmitoyl acyltansferases reside. The hydroxylamine (HA)-dependent biotin switch, a reverse labeling technique for palmitoylated cysteines (see Fig. 3*A*), was used to determine the ratio of endogenous palmitoylated *versus* total H-Ras in HAECs. Reducing all reversibly oxidized cysteines with DTT and hydrolyzing the thioester bonds with HA, all 5 cysteines on H-Ras were labeled (Fig. 3*B*, *lane 1*). Using HA alone, only palmitoylated cysteines were selectively labeled (Fig. 3*B*, *lane 2*). Pull-down of the labeled protein with streptavidin beads followed by SDS-PAGE and immunoblotting against H-Ras showed that only 31% of total endogenous H-Ras in HAECs was palmitoylated (Fig. 3, *B* and *C*).

**FIGURE 4.**
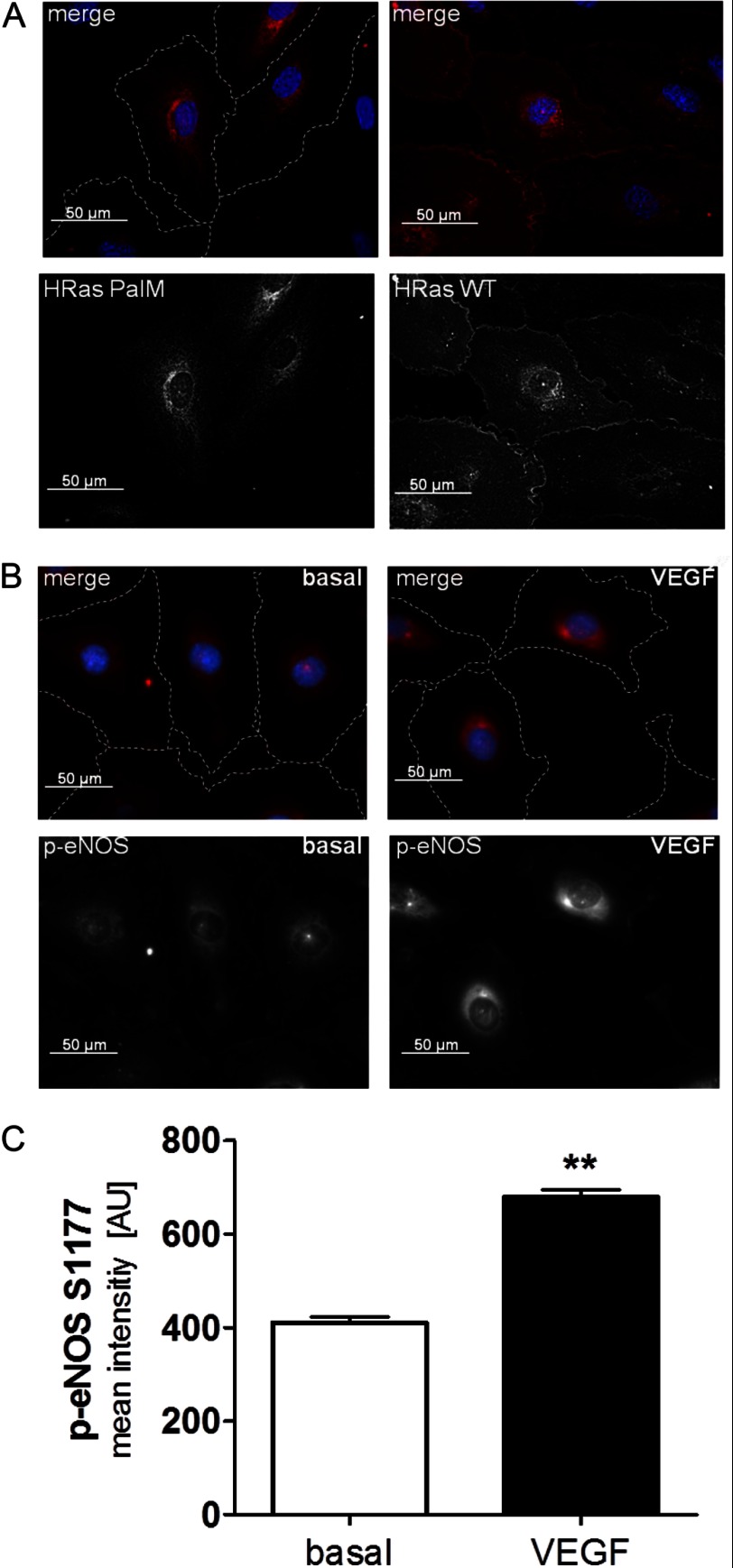
**Subcellular distribution of H-Ras and phospho-eNOS after VEGF stimulation.** *A*, *left*, the palmitoylation-deficient H-Ras PalM (C181S/C184S) localizes predominantly to endomembranes. *Right*, adenoviral H-Ras WT localizes to the plasma membrane and the perinuclear region in EC. For detection of the overexpressed H-Ras WT and PalM, an antibody against the XPRESS affinity tag was used. Pictures were taken by confocal microscopy. Cell borders are *outlined* with a *white dashed line* for H-Ras PalM. *B*, eNOS phosphorylation at serine Ser-1177 is increased in the perinuclear region after 30-min VEGF stimulation. HAECs were starved overnight, treated with VEGF (30 min, 50 ng/ml), and subsequently fixed and stained for eNOS (*phospho S1177*, *red*) and cell nuclei (DAPI, *blue*). Staining was performed as described under “Materials and Methods.” Pictures were taken at ×400, using epifluorescence. Cell borders are *outlined* with a *white dashed line* in the *merged pictures. C*, mean fluorescent intensity of staining, expressed in arbitrary units (AU), for eNOS phosphorylation at serine 1177 was measured in the perinuclear region in 35–50 cells/experiment under the same conditions using NIS-Elements software. Unpaired Student's *t* test was used (*n* = 2). *Error bars*, S.E. **, *p* < 0.01.

Because the majority of H-Ras in ECs is not palmitoylated, it is expected to localize exclusively to endomembranes (29–31). Accordingly, HAECs moderately overexpressing H-Ras WT showed both PM and endomembrane localization (Fig. 4*A*, *right*). Mutation of both palmitoylation sites C181S and C184S (H-Ras PalM), as shown previously in NIH3T3 (31) and MDCK cell lines (30), causes localization of the mutant solely to endomembranes in HAECs (Fig. 4*A*, *left*). VEGF-mediated activation of eNOS (phosphorylation at Ser-1177 after 30 min) primarily occurred in the perinuclear region, as shown in Fig. 4, *B* and *C*, also indicating endomembrane signaling of eNOS. Thus, the H-Ras PalM mutant serves as a model to study H-Ras function at this subcellular compartment because other proteins necessary in angiogenic signaling, including eNOS, show similar subcellular localization and activation.

##### Endomembrane-delimited H-Ras Is Sufficient to Mediate VEGF-induced Endothelial Cell Migration

**FIGURE 5.**
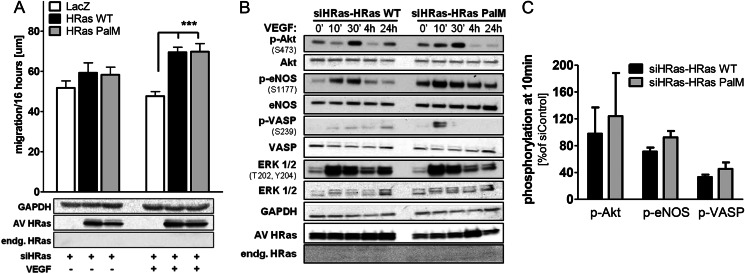
**Endomembrane H-Ras is mediating VEGF-induced migration by activating the PI3K/Akt/eNOS signaling cascade.** *A*, in H-Ras-depleted ECs, reconstitution of endogenous H-Ras with either adenoviral H-Ras WT or endomembrane H-Ras PalM resulted in recovery of migration in response to VEGF. After overnight starvation, a wound healing assay was performed with H-Ras-depleted HAECs using VEGF (50 ng/ml) as a stimulus. Two-way ANOVA and Bonferroni's post-test were used (*n* ≥ 5). *Error bars*, S.E. ***, *p* < 0.001. *B*, reconstitution of endogenous H-Ras with either adenoviral H-Ras WT or H-Ras PalM resulted in partial recovery of the key proangiogenic signaling cascade Akt/eNOS in response to VEGF. HAECs treated with siH-Ras and reconstituted with H-Ras WT or PalM were stimulated after overnight starvation for 0 min, 10 min, 30 min, 4 h, and 24 h with VEGF (50 ng/ml). *C*, densitometric quantification of HAECs treated with siH-Ras and reconstituted with H-Ras WT or H-Ras PalM at 10-min VEGF stimulation. All values were standardized to GAPDH as a loading control and normalized to siC-LacZ at 0 min. *n* ≥ 3. *Error bars*, S.E.

As demonstrated in Fig. 2*A*, knockdown of endogenous H-Ras inhibits EC migration and signaling caused by VEGF, confirming that H-Ras is required for proangiogenic signaling. Reconstitution of H-Ras-depleted HAECs with the H-Ras PalM mutant restored the migratory phenotype (Fig. 5*A*). In addition, restoration of the proangiogenic signaling cascade PI3K/Akt/eNOS/VASP also occurred by overexpressing H-Ras PalM (Fig. 5, *B* and *C*), indicating that H-Ras at the endomembrane can fully reconstitute proangiogenic signaling by VEGF for induced ^•^NO production and EC migration.

##### Endomembrane H-Ras Is Sufficient for VEGF-induced Akt and eNOS Phosphorylation and ^•^NO Production

**FIGURE 6.**
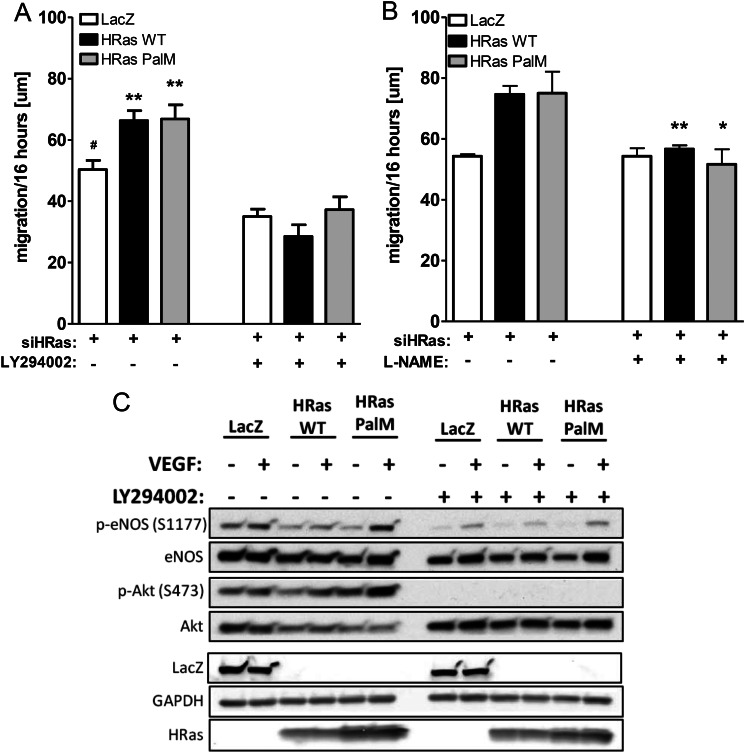
**Endomembrane H-Ras activates ^•^NO production by activating the PI3K/Akt/eNOS signaling cascade.** *A*, H-Ras-mediated EC migration was inhibited by inhibition of PI3K. H-Ras depleted and reconstituted with either LacZ, H-Ras WT, or H-Ras PalM HAECs were stimulated with VEGF (50 ng/ml), and an EC wound healing assay was performed. ECs were treated with either DMSO or the PI3K inhibitor LY294002 (5 μm) during migration. One-way ANOVA and Dunnett's post-test were used (*n* ≥ 4). *Error bars*, S.E. **, *p* < 0.01, treatment *versus* no treatment; *, *p* < 0.05, reconstitution with LacZ *versus* H-Ras WT or PalM. *B*, H-Ras-mediated EC migration was reduced by inhibiting eNOS with l-NAME. The wound healing assay was performed as described above, and cells were treated with l-NAME (1 mm) for the duration of the assay. Unpaired Student's *t* test was used (*n* ≥ 3). *Error bars*, S.E. **, *p* < 0.01; *, *p* < 0.05, treatment *versus* no treatment. *C*, H-Ras-mediated proangiogenic signaling was reduced by inhibition of PI3K. H-Ras-depleted and -reconstituted HAECs were preincubated with LY294002 (10 μm) and then stimulated with VEGF (50 ng/ml, 30 min). A representative Western blot is shown.

As shown in Fig. 5, reconstitution of H-Ras-depleted HAECs with the endomembrane-localized H-Ras PalM, restored Akt Ser-473 and eNOS Ser-1177 phosphorylation in response to VEGF. Consistent with eNOS activation, the enhanced phosphorylation of VASP at Ser-239 indicates an increase in PKG caused by eNOS-derived ^•^NO (Fig. 2, *C* and *D*). Although overexpression of adenoviral H-Ras WT results in increased ERK 1/2 phosphorylation, the main portion of VEGF-mediated activation of the Raf/MEK/ERK cascade is mediated by PKC in EC (19, 20). Conversely, we found that blocking PKC with Gö6983 in HAECs had no effect on VEGF-mediated Akt and eNOS phosphorylation (data not shown). In contrast, inhibition of PI3K with the specific inhibitor LY 294002 prevented VEGF-induced Akt and eNOS phosphorylation as well as EC migration mediated by not only WT but also H-Ras PalM (Fig. 6, *A* and *C*), demonstrating the importance of PI3K/Akt/eNOS signaling downstream of endomembrane H-Ras. In addition, treatment of cells with the eNOS inhibitor l-NAME prevented VEGF-induced migration in HAECs depleted of H-Ras and reconstituted with either H-Ras WT or PalM (Fig. 6*B*), demonstrating that endomembrane H-Ras is sufficient to explain VEGF-induced ^•^NO-mediated EC migration.

##### VEGF Activates Endomembrane-delimited H-Ras

**FIGURE 7.**
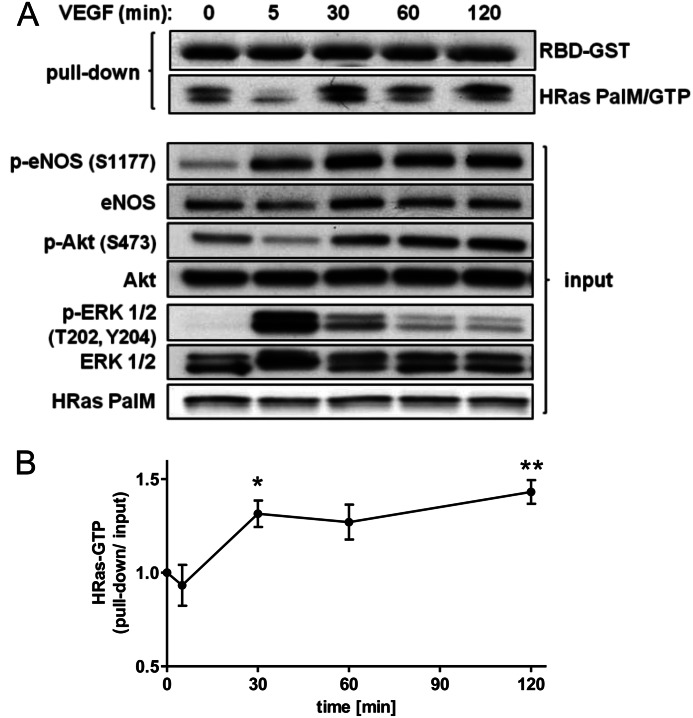
**Endomembrane H-Ras is activated by VEGF stimulation.** *A*, HAECs depleted of endogenous H-Ras and reconstituted with H-Ras PalM were starved overnight and treated for increasing periods of time with VEGF (50 ng/ml). Cells were harvested in RBD-GST-containing lysis buffer, and active H-Ras (H-Ras/GTP) was pulled down using glutathione-agarose beads. Coomassie-stained RBD-GST was shown to demonstrate equal loading of RBD-GST. Representative blots of pull-down and input were shown. *B*, pulled down H-Ras PalM quantified using ImageJ and normalized to H-Ras input. One-way ANOVA and Dunnett's post-test were used (*n* ≥ 4). *Error bars*, S.E. *, *p* < 0.05; **, *p* < 0.01.

As demonstrated in Figs. 5 and 6, the palmitoylation-deficient mutant H-Ras PalM is fully capable of mediating VEGF-induced signaling via PI3K/Akt/eNOS and EC migration. However, only GTP-bound H-Ras interacts with its downstream effectors mediating signaling. To support our hypothesis, we measured the ratio of GTP-bound H-Ras to total H-Ras in HAECs stimulated with VEGF over a time course. HAECs depleted of endogenous H-Ras and reconstituted with H-Ras PalM show an increase in endomembrane H-Ras/GTP starting at 30 min with activity persisting over a time period of 2 h. Unlike EGF, which causes H-Ras activation in HEK 293T and HeLa cells within seconds to 5 min (13, 32), VEGF causes a late and sustained activation of endomembrane H-Ras in endothelial cells, mirroring Akt and eNOS phosphorylation/activity (Fig. 7).

## DISCUSSION

Here we define a novel role for endomembrane H-Ras in VEGF-induced EC migration. Utilizing a knockdown/reconstitution approach, we show that palmitoylation-deficient H-Ras localized at the endomembranes is preferentially activated by VEGF and fully capable of mediating proangiogenic signaling via PI3K-dependent phosphorylation and activation of Akt and eNOS, enabling EC migration.

Ras isoforms are essential components of growth factor signaling. Although expressed ubiquitously, mRNA levels of the various isoforms appear to be regulated both spatially and temporally. K-Ras knock-out animals are embryonic lethal, whereas N-Ras- and H-Ras-deficient mice show no obvious developmental abnormality (33). However, the importance of H-Ras in post-natal EC angiogenic signaling is undeniable, given its role in differentiating vascular progenitors into ECs stimulated by VEGF (34) and development of capillary-like structures (35). Finally, our results show clearly the essential role of H-Ras in EC migration. Its unique function is further supported, in that depletion of N-Ras shows no impairment of EC migration in response to VEGF (Fig. 2*B*). The Ras isoform expression profiles of ECs (Fig. 1*A*) suggest that our model of endomembrane H-Ras signaling in HAECs may also translate into microvascular ECs. HUVECs, however, represent fetal ECs, which express N-Ras as the predominant isoform and exhibit comparably higher levels of K-Ras than aortic and microvascular ECs (Fig. 1*A*), suggesting differences in Ras signaling.

Serban *et al.* (16) characterized the role of the two main downstream H-Ras effectors, utilizing constitutively active H-Ras mutants that bind to either Raf or the PI3K catalytic subunit p110. They showed that interaction with Raf alone was capable of mediating angiogenesis, whereas interaction with p110 isoforms increased survival signaling and vascular permeability. In contrast, we modestly overexpressed a non-constitutively active H-Ras (≤10-fold over endogenous H-Ras) that requires the same activating and inactivating factors as the endogenous GTPase. Thus, our studies may better reflect physiological activation of H-Ras by VEGF and proangiogenic downstream effects.

Unlike other growth factors, the requirement for H-Ras in VEGF-mediated activation of the ERK MAPK cascade in ECs is still debated. Although VEGF receptor activation promotes Ras activity via association with adaptor protein Grb2 (18, 19, 36), we and others (20, 37, 38) found that VEGF-induced ERK phosphorylation and downstream signaling strongly depends on PKC activation. Akt and eNOS phosphorylation are, however, unaffected by the PKC inhibitor Gö6983 (data not shown). In support of Ras-mediated Akt signaling in angiogenesis, mice with a defective RBD in p110 show branching defects in their vascular lymphatic system and loss of Akt activation in response to growth factors in cultured mouse embryonic fibroblasts (39).

Several studies investigated eNOS activity and ^•^NO production in response to VEGF at different membrane locations, revealing that eNOS at the PM is sensitive to early calcium-dependent activation, whereas eNOS at the endomembranes shows a delayed response associated with phosphorylation at Ser-1177 by Akt (25, 40–42).

H-Ras interaction with cellular membranes is regulated by C-terminal lipid modifications. Farnesylation at cysteine 186 allows H-Ras to bind to endomembranes but not PM as our (Fig. 4*A*) and other studies (6, 29, 30) have shown. Single (either Cys-181 or Cys-184) or double *S*-palmitoylation, however, does lead to PM localization via Golgi vesicular transport. Interestingly, single *S*-palmitoylated N-Ras and H-Ras (C181S or C184S mutants) show faster depalmitoylation, resulting in shorter PM interaction compared with double *S*-palmitoylated H-Ras WT (30). Utilizing the palmitoylation-deficient mutant H-Ras PalM, as a model for H-Ras endomembrane signaling, we uncovered a novel VEGF to H-Ras link that omits activation of the small GTPase at the PM and is sufficient for signaling PI3K/Akt/eNOS-mediated migration. Studies in various other cell types reported that wild type and constitutively active endomembrane H-Ras increase ERK signaling upon growth factor stimulation (31, 43, 44). Our studies in adult ECs stimulated with VEGF suggest, however, that ERK is stimulated by PKC, and endomembrane-localized H-Ras is responsible for stimulation of proangiogenic signaling via PI3K/Akt/eNOS. Indeed, our experiments using knockdown/H-Ras PalM reconstitution repeatedly showed increased Akt and eNOS phosphorylation (see Figs. 5 (*B* and *C*) and 6*C*), demonstrating that VEGF can increase eNOS activity via H-Ras at the endomembranes, where it has been reported to cause local oxidative protein modifications, including *S*-nitrosylation (22, 45). Nevertheless, the signaling mechanism from PM-bound GF receptors to the endomembranes is still in question and may vary in different cell types. Studies in EGF-stimulated COS-1 cells identified the calcium-sensitive Ras GRP1 guanosine exchange factor as an H-Ras activator at the endomembranes and provided evidence that growth factor stimulation of Src/PLCγ activates guanosine exchange factors through increase in local calcium and DAG (12, 46, 47).

By linking endomembrane H-Ras to VEGF-mediated EC migration, our studies have identified a novel mechanism for eNOS activation in adult ECs. We were able for the first time to assign endomembrane H-Ras an active part in EC signaling, relating it to the key PI3K/Akt/eNOS angiogenic signaling pathway that results in nitric oxide production and EC migration.

## Footnotes

2 The abbreviations used are:

RBD: Ras-binding domain
PM: plasma membrane
l-NAME: *N*^G^-nitro-l-arginine methyl ester
EC: endothelial cell
HAEC: human aortic endothelial cell
eNOS: endothelial nitric-oxide synthase
VASP: vasodilator-stimulated phosphoprotein
HA: hydroxylamine
HUVEC: human umbilical vein endothelial cell
ANOVA: analysis of variance.
